# Supplementary material for: Clustering of SARS-CoV-2 membrane proteins in lipid bilayer membranes
Source: PLoS Comput Biol. 2026 Apr 27;22(4):e1014229. doi: 10.1371/journal.pcbi.1014229 (PMC13148779; doi:10.1371/journal.pcbi.1014229)
Supplement: S1 Appendix — (PDF) [file pcbi.1014229.s001.pdf]

S1 Appendix. Nonlinear form of continuum model and conversion to linearity. With our complete free energy shown in Eq. 17, the corresponding variational derivative can be seen in Eq. 18.

$$\mathcal{F} = \int_{\mathcal{S}} \left[\frac{k_{\mathrm{B}}T}{a^2} \Big[(1-\rho)\ln(1-\rho) + \rho\ln(\rho) \Big] + \frac{\epsilon_m}{2a^2}\rho - \frac{\epsilon_m}{2a^2}\rho^2 + \frac{\epsilon_m}{4}\left|\nabla\rho\right|^2 \right] dxdy$$
$$= \int_{\mathcal{S}} f(\rho, \nabla\rho)\, dxdy \quad (17)$$

$$\frac{\delta\mathcal{F}}{\delta\rho} = \frac{\partial f}{\partial\rho} - \nabla\cdot\left[\frac{\partial f}{\partial(\nabla\rho)}\right] = \frac{k_{\mathrm{B}}T}{a^2}\left[\ln(\rho) - \ln(1-\rho) + \frac{\tilde{\epsilon_m}}{2} - \tilde{\epsilon_m}\rho - \frac{\tilde{\epsilon_m}}{2}\tilde{\nabla}^2\rho\right] \quad (18)$$

Tildes designate nondimensionalization, as seen with $\tilde{\epsilon}_m = \frac{\epsilon_m}{k_{\mathrm{B}}T}$ and $\tilde{\nabla} = a\nabla$. As a result, the nondimensionalized conservation equation becomes:

$$\frac{\partial\rho}{\partial t} = \frac{A}{k_{\mathrm{B}}T}\left[\rho\tilde{\nabla}^2\left(\frac{\delta\mathcal{F}}{\delta\rho}\right) + \tilde{\nabla}\rho\cdot\tilde{\nabla}\left(\frac{\delta\mathcal{F}}{\delta\rho}\right)\right]$$
$$= \frac{A}{a^2}\left[\left(\frac{1}{\rho(1-\rho)} - \tilde{\epsilon}_m\right)\rho\tilde{\nabla}^2\rho - \frac{\tilde{\epsilon}_m}{2}\rho\tilde{\nabla}^4\rho + \left(\frac{1}{(1-\rho)^2} - \tilde{\epsilon}_m\right)\left|\tilde{\nabla}\rho\right|^2 - \frac{\tilde{\epsilon}_m}{2}\tilde{\nabla}\rho\cdot\tilde{\nabla}\left(\tilde{\nabla}^2\rho\right)\right] \quad (19)$$

With the form of Eq. 7, where $\delta\rho << 1$, the following is true and converts Eq. 19 to linearity.

$$\left(\frac{1}{\rho(1-\rho)} - \tilde{\epsilon}_m\right)\rho\tilde{\nabla}^2\rho = \left(\frac{1}{\rho^*(1-\rho^*)} - \tilde{\epsilon}_m\right)\rho^*\tilde{\nabla}^2\rho \quad (20)$$

$$\frac{\tilde{\epsilon}_m}{2}\rho\tilde{\nabla}^4\rho = \frac{\tilde{\epsilon}_m}{2}\rho^*\tilde{\nabla}^4\rho \quad (21)$$

$$\left(\frac{1}{(1-\rho)^2} - \tilde{\epsilon}_m\right)\left|\tilde{\nabla}\rho\right|^2 = 0 \quad (22)$$

$$\frac{\tilde{\epsilon}_m}{2}\tilde{\nabla}\rho\cdot\tilde{\nabla}\left(\tilde{\nabla}^2\rho\right) = 0 \quad (23)$$

As a result, the linearized density evolution equation can be seen in Eq. 9. Plugging Eq. 8 into the final linear form leads to the dispersion relation shown in Eq. 11. From here, the maximum wavevector (q_{max}) and growth rate ($\omega_{0,max}$) can be defined accordingly for $\tilde{\epsilon}_m > 0$.

$$q_{max} = 0, \pm\sqrt{1 - \frac{1}{\tilde{\epsilon}_m\rho^*(1-\rho^*)}} \quad (24)$$

$$\omega_{0,max} = \frac{A\rho^*\left(\frac{1}{\rho^*(1-\rho^*)} - \tilde{\epsilon}_m\right)^2}{2a^2\tilde{\epsilon}_m} = \frac{A\rho^*\tilde{\epsilon}_m q_{max}^4}{2a^2} \quad (25)$$

Solving Eq. 24 for $\tilde{\epsilon}_m$ leads to Eq. 15, while using $d = \frac{2\pi a}{q_{max}}$ with Eq. 24 leads to Eq. 16. Additionally the curve shown in Fig 4c and S11 Fig, is Eq. 25 for the corresponding density fraction.
