# Supplementary material for: Clustering of SARS-CoV-2 membrane proteins in lipid bilayer membranes
Source: PLoS Comput Biol. 2026 Apr 27;22(4):e1014229. doi: 10.1371/journal.pcbi.1014229 (PMC13148779; doi:10.1371/journal.pcbi.1014229)
Supplement: S2 Appendix — (PDF) [file pcbi.1014229.s002.pdf]

S2 Appendix. Approximating the contribution of line tension in effective interaction energy from a discrete protein lattice to a continuum. To account for the contribution of line tension in effective interaction energy, the energy difference for proteins far apart and close together is determined and compared to the form shown in Eq. 12. As seen in S2 Fig panel a, we start with a square protein lattice, where sites can be occupied with protein or membrane and are a distance a apart (where a is equivalent to the approximate width of an M protein). Initially, two M proteins are spread apart such that they do not experience any nearest neighbor interactions. The corresponding energy for a general system with only two proteins can be seen below, where N is the total number of site-site interactions.

$$\mathcal{E}_i = (N-8)\epsilon_{mem-mem} + 8\epsilon_{m-mem} \tag{26}$$

Next, in S2 Fig panel b, these proteins are moved together such that they experience nearest neighbor interactions. As a result, the total energy of the system ($\mathcal{E}_f$) is seen in Eq. 27.

$$\mathcal{E}_f = (N-7)\epsilon_{mem-mem} + 6\epsilon_{m-mem} + \epsilon_{m-m} \tag{27}$$

Thus, the energy difference ($\Delta\mathcal{E}_f$) can be found in Eq. 28, where the factor of z is a result of adding the contribution from each axis (z defines the number of nearest neighbors, equal to four with a square lattice).

$$\Delta\mathcal{E} = \mathcal{E}_f - \mathcal{E}_i = z\left(\epsilon_{mem-mem} + \epsilon_{m-m} - 2\epsilon_{m-mem}\right) = \epsilon_m \tag{28}$$

Now, since the energy difference from this discrete lattice is equivalent to the effective interaction energy ϵ_m, the energetic benefit of bringing these two proteins together can be equated to this effective interaction energy. Initially, the contribution from line tension for two separate M proteins is shown below, proportional to the surface area, where γ_m is the line tension shown in Eq. 2. Proteins are considered square due to the square lattice chosen in our derivation and numerical solution. The negative sign keeps convention consistent with $\epsilon_m > 0$ representing attraction.

$$\mathcal{E}_i = -8a\gamma_m \tag{29}$$

As the square proteins are brought together, the surface area changes to a larger rectangle with a surface area of $6a$, and direct protein-protein interactions play a more prominent role as an effective oligomerization energy.

$$\mathcal{E}_f = -6a\gamma_m + \epsilon_{\text{olig}} \tag{30}$$

With Eq. 29 and Eq. 30 the energy difference ($\Delta\mathcal{E}$) can be determined and compared to the effective interaction energy.

$$\Delta\mathcal{E} = \epsilon_{\text{olig}} + 2a\gamma_m = \epsilon_m \tag{31}$$

From this method, the contribution from thinning induced line tension in effective interaction energy can be estimated.
