# Supplementary figures and images for: Clustering of SARS-CoV-2 membrane proteins in lipid bilayer membranes
Source: PLoS Comput Biol. 2026 Apr 27;22(4):e1014229. doi: 10.1371/journal.pcbi.1014229 (PMC13148779; doi:10.1371/journal.pcbi.1014229)

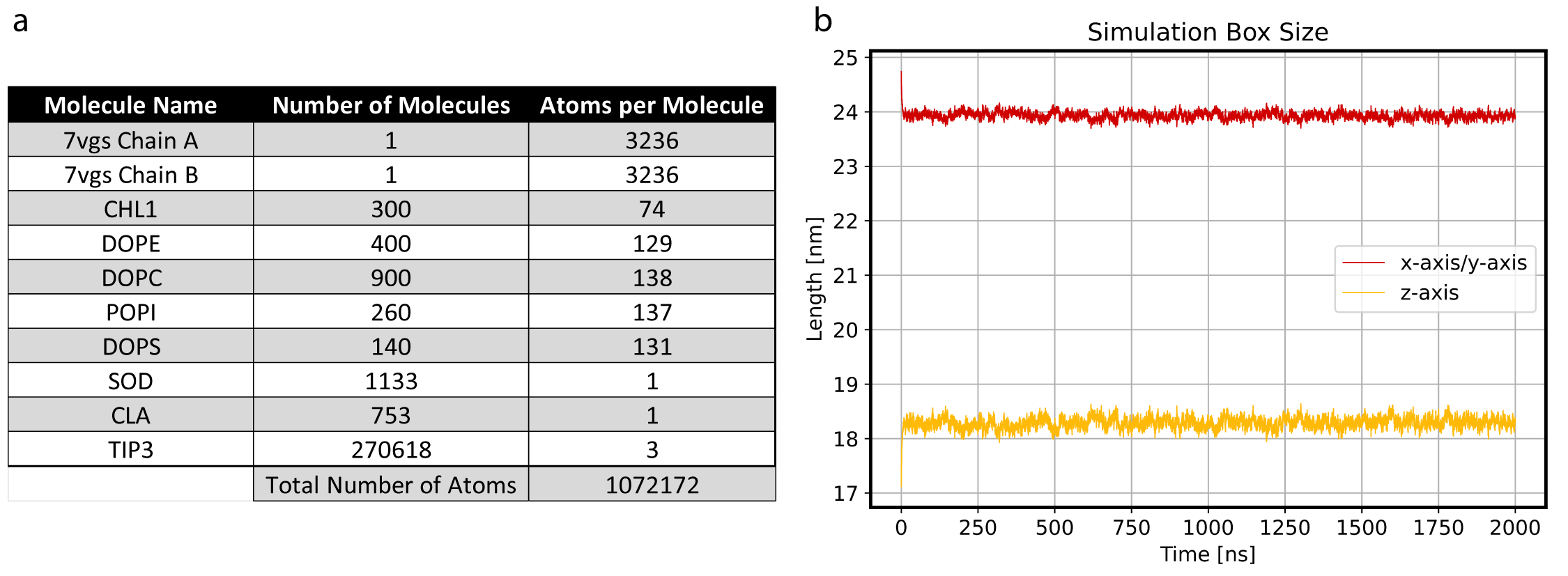

Supplement: S1 Fig — (a) Since the leaflets are symmetric, the number of molecules for a corresponding lipid type in a leaflet is half the system-wide value, adding to 1000 lipid molecules per leaflet. (b) After a quick change in the length along each axis within the first few nanoseconds, the box remains stable throughout the simulation. (TIF) [file pcbi.1014229.s003.tif]

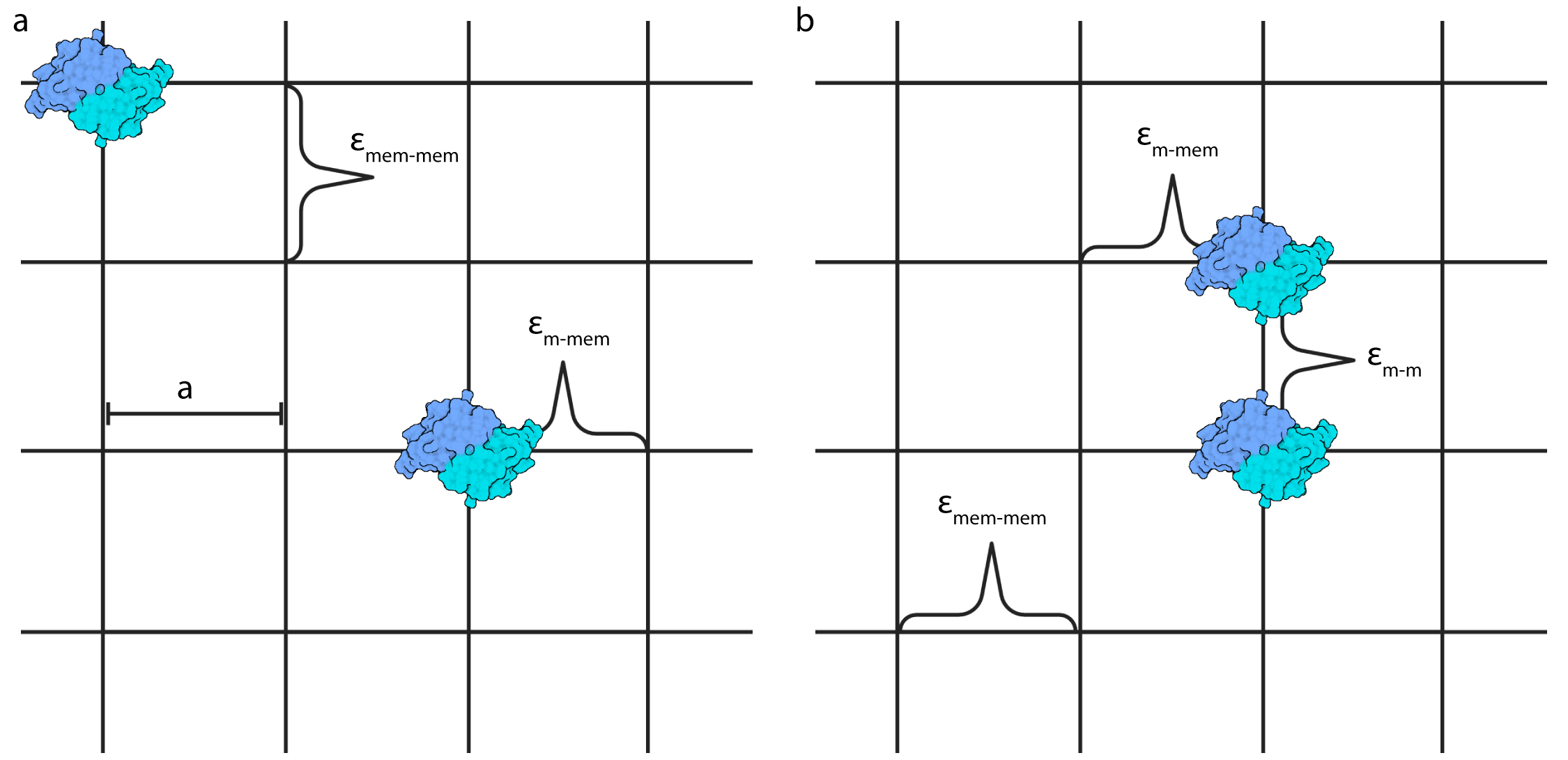

Supplement: S2 Fig — The discrete protein lattice, with distance between lattice sites equivalent to the approximate width of the protein a, shows the two prominent types of site-site interactions when two proteins are not nearest neighbors: ϵmem−mem and ϵm−mem. (b) As these proteins become nearest neighbors, ϵm−m encompasses direct interactions between proteins. Converting this representation for a system of randomly distributed proteins to a continuum results in the described continuum model. Created with BioRender.com (https://biorender.com/gxjhs1k, https://biorender.com/bkbr3e1). (TIF) [file pcbi.1014229.s004.tif]

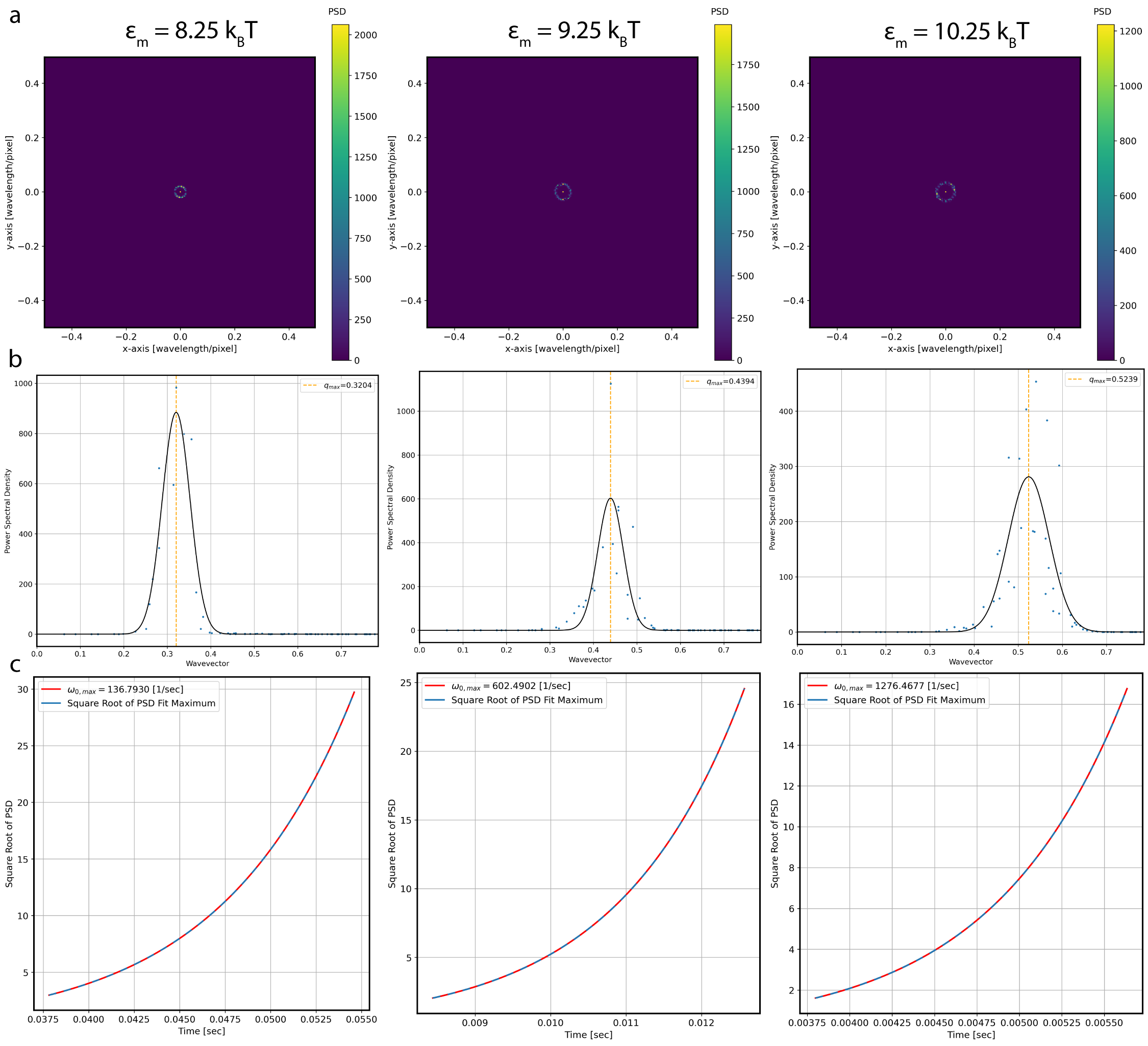

Supplement: S3 Fig — (a) Power spectra are shown for the three images displayed in Fig 4a, where the colorbar displays power spectrum density (PSD) dependent on wavelength per pixel. The radius of the prominent ring is the maximum wavevector. (b) After radially binning from the center of the spectra and averaging the PSD of non-unique radii, the radial profile as a function of the wavevector is shown for each effective interaction energy (wavevector is 2πadl times wavelength per pixel). Blue dots represent the average PSD for every distance from the center, while the black curve shows the corresponding gaussian fit with the maximum wavevector shown as the dotted orange line. (c) The square root of the PSD fit maxima for each measured time is shown with the dotted blue line, while the exponential growth fit is shown in red. The growth rate used for each of these plots is the corresponding maximum growth rate. (TIF) [file pcbi.1014229.s005.tif]

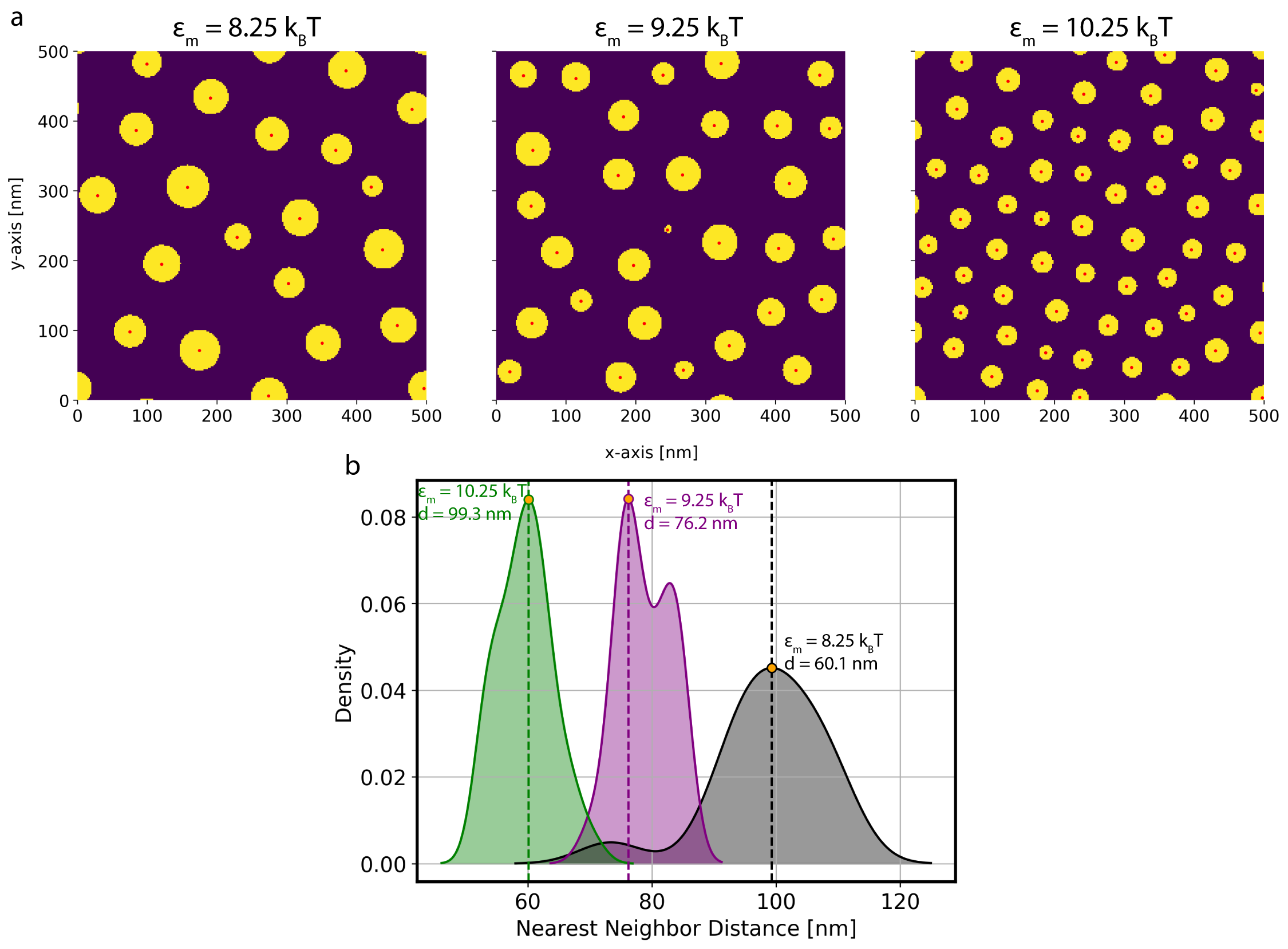

Supplement: S4 Fig — (a) Plot of the thresholded simulations at the cut-off time shown in Fig 4b, where regions with protein are shown in yellow and cluster centroids are shown in red. (b) Kernel density estimate for each of the three images in black (ϵm=8.25 kBT), purple (ϵm=9.25 kBT), and green (ϵm=10.25 kBT), where the dotted line displays the nearest neighbor distance in which there is a maximum (d). Each of these distances are shown as orange dots in Fig 4d. (TIF) [file pcbi.1014229.s006.tif]

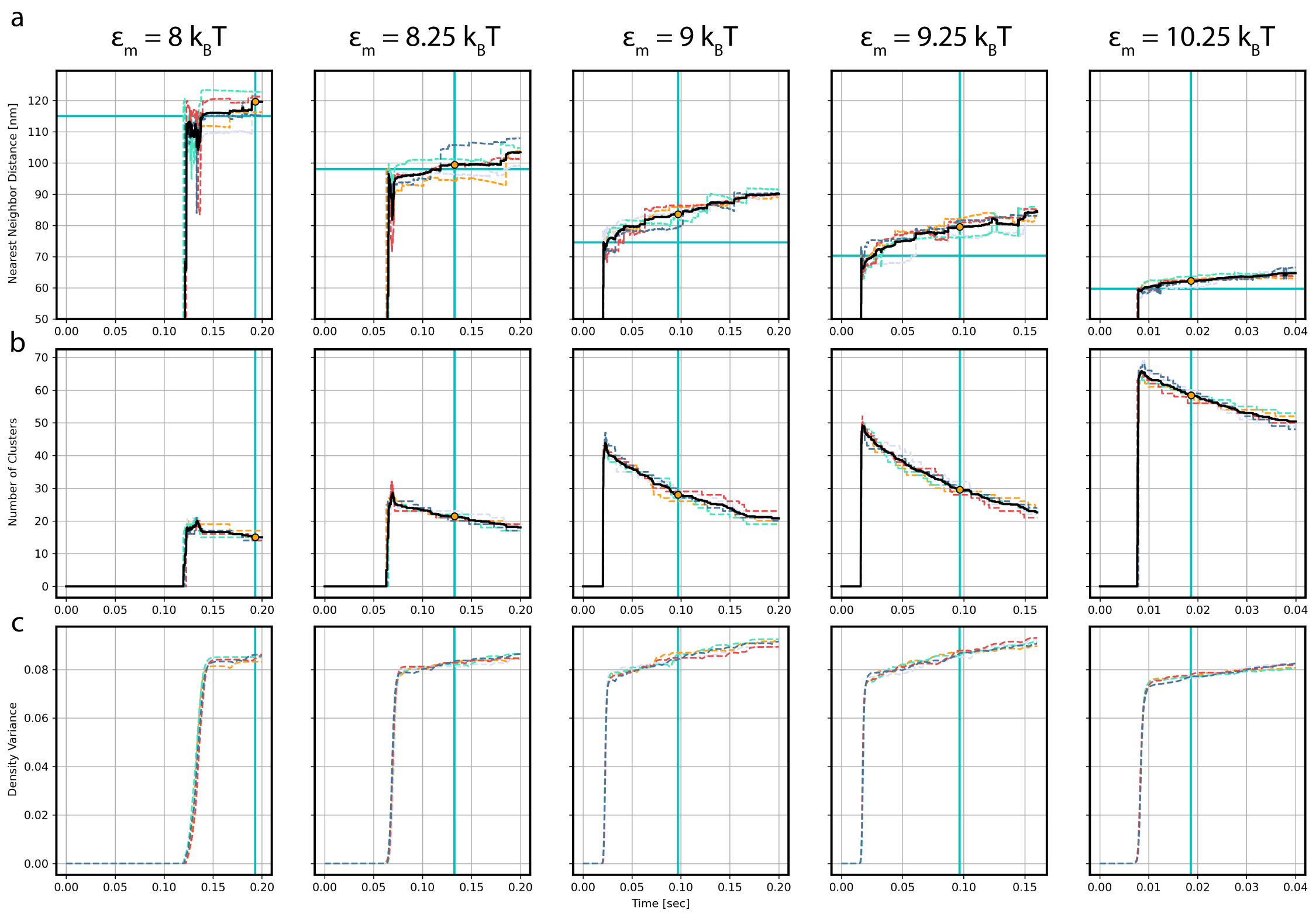

Supplement: S5 Fig — Each replicate is shown as a dotted line of variable color, where the corresponding average of all replicates is shown as a black line for (a) and (b). Vertical cyan lines represent the cut-off time calculated by finding the time of minimal slope for the average nearest neighbor evolution curve. This time changes for each interaction energy, where t8 = 0.193 s, t8.25 = 0.13265 s, t9 = .0969 s, t9.25 = .09668 s, and t10.25 = .01863 s. The average value in (a) and (b) at the cut-off time is displayed with an orange dot. For (a), the horizontal cyan line represents the analytically predicted nearest neighbor distance. In (c), the slight fluctuations or bumps in density variance are a result of the gradually decreasing number of clusters stemming from Ostwald ripening shown in (b) for each interaction energy. None of these quantities were gathered until ρmax>0.9. (TIF) [file pcbi.1014229.s007.tif]

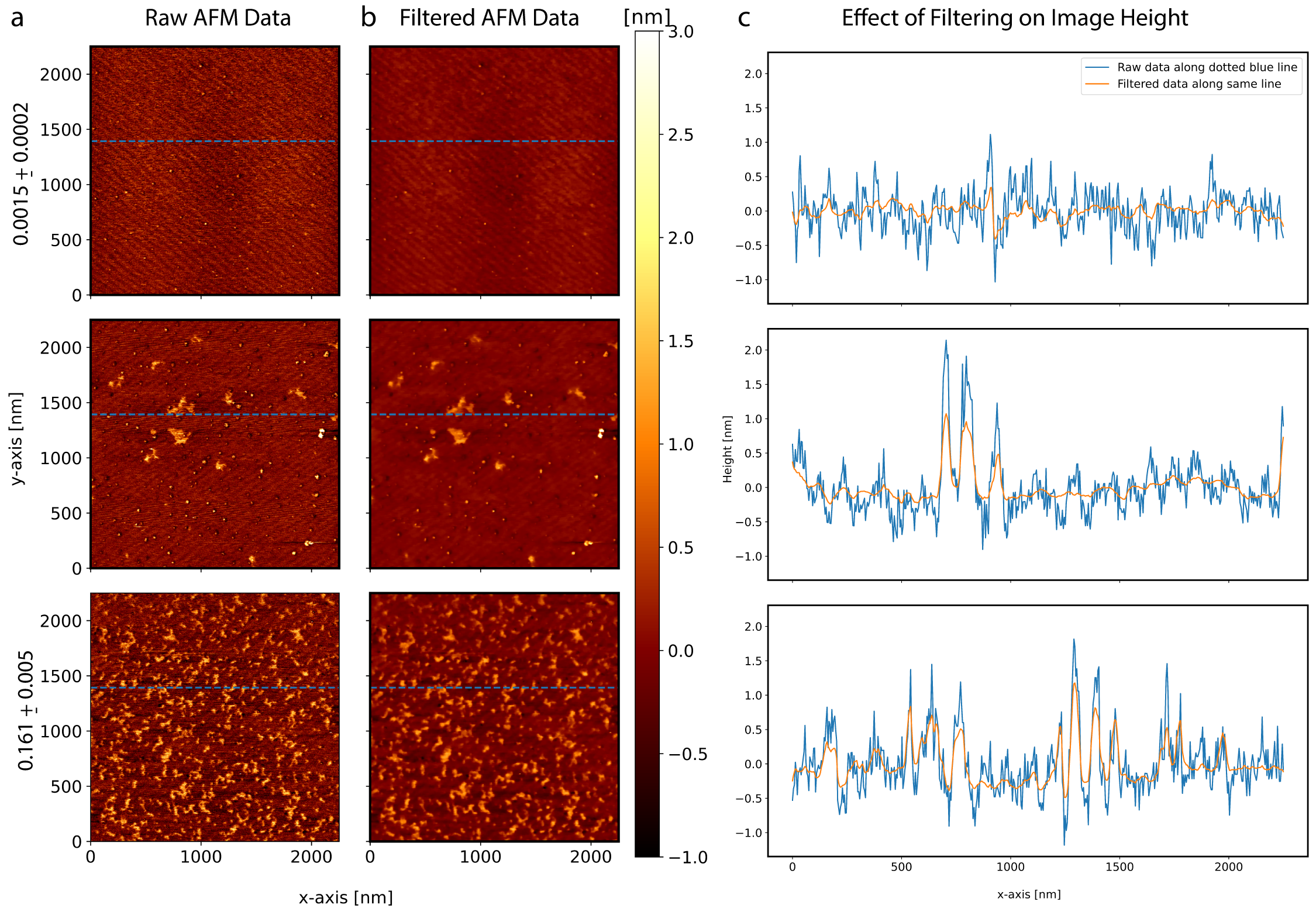

Supplement: S6 Fig — (a) The raw AFM data at the three different protein area coverages shown in Fig 2d. (b)-(c) This AFM data was filtered with total variation denoising using split-Bregman optimization, with a denoising weght of one and a tolerance of 1 × 10^−5^. Height values for the cross-section shown in (a) and (b) as a dotted blue line are displayed in (c) for comparison between the raw and filtered data. (TIF) [file pcbi.1014229.s008.tif]

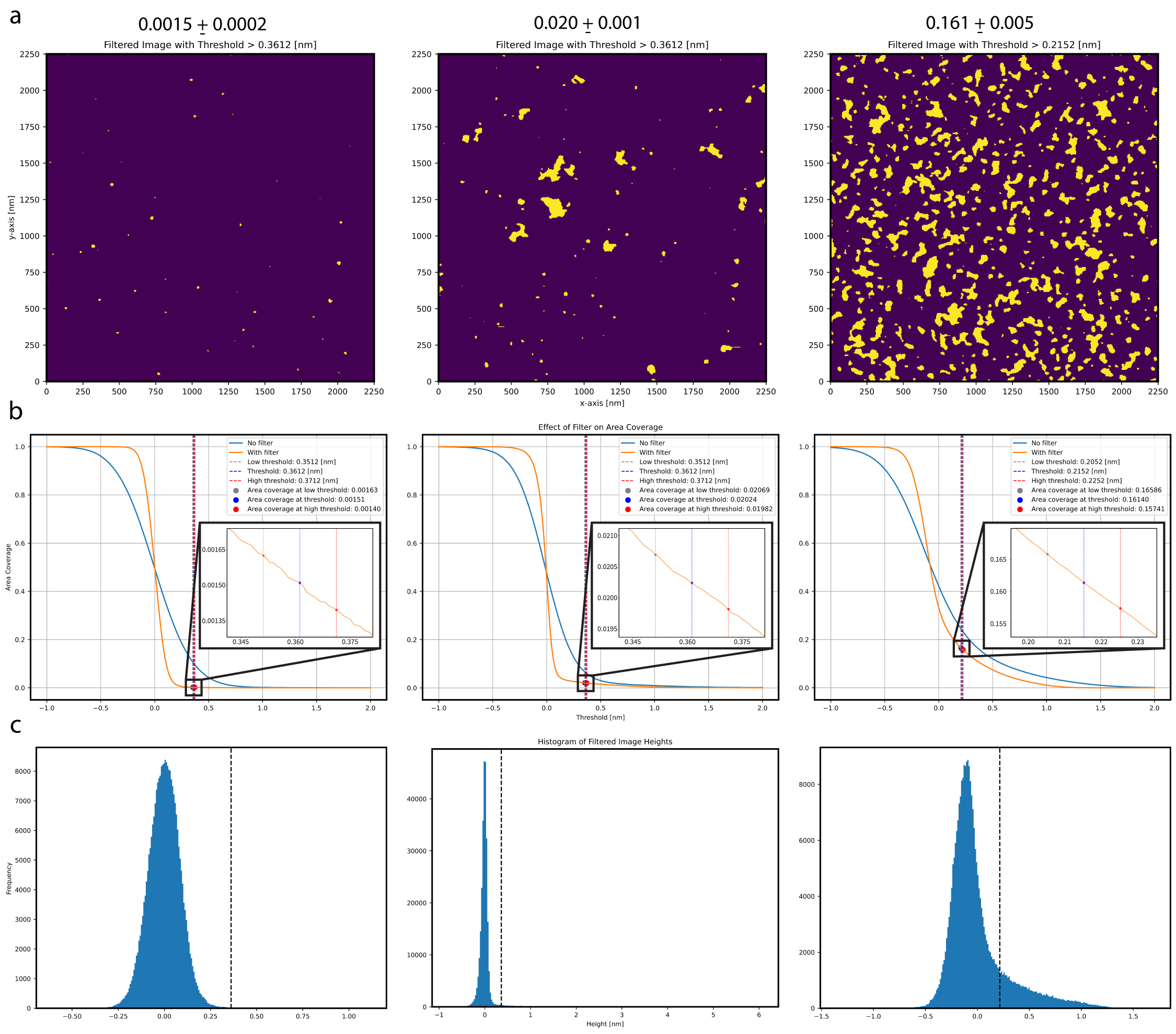

Supplement: S7 Fig — Height thresholds for the two highest area coverages were found using Otsu’s method, where the threshold can be found above each image in (a). Due to the low protein density in the leftmost image, its threshold was chosen to match that of the middle panel. (b) Protein area coverage is shown as a function of threshold height, where the used value is shown as a dotted vertical blue line. As the filter is applied, the curve gets closer to a step function, as expected for a transmembrane protein. The dotted grey line represents the higher bound on area coverage when considering AFM vertical resolution, while the dotted red line signifies the reverse, leading to the error bars in (a). Insets highlighting the change in area coverage near the chosen threshold heights are displayed for each system. Lastly, (c) displays a histogram of filtered image heights, with the threshold as a dotted black line. Note that Otsu’s method loses effectiveness as the data becomes more Gaussian. (TIF) [file pcbi.1014229.s009.tif]

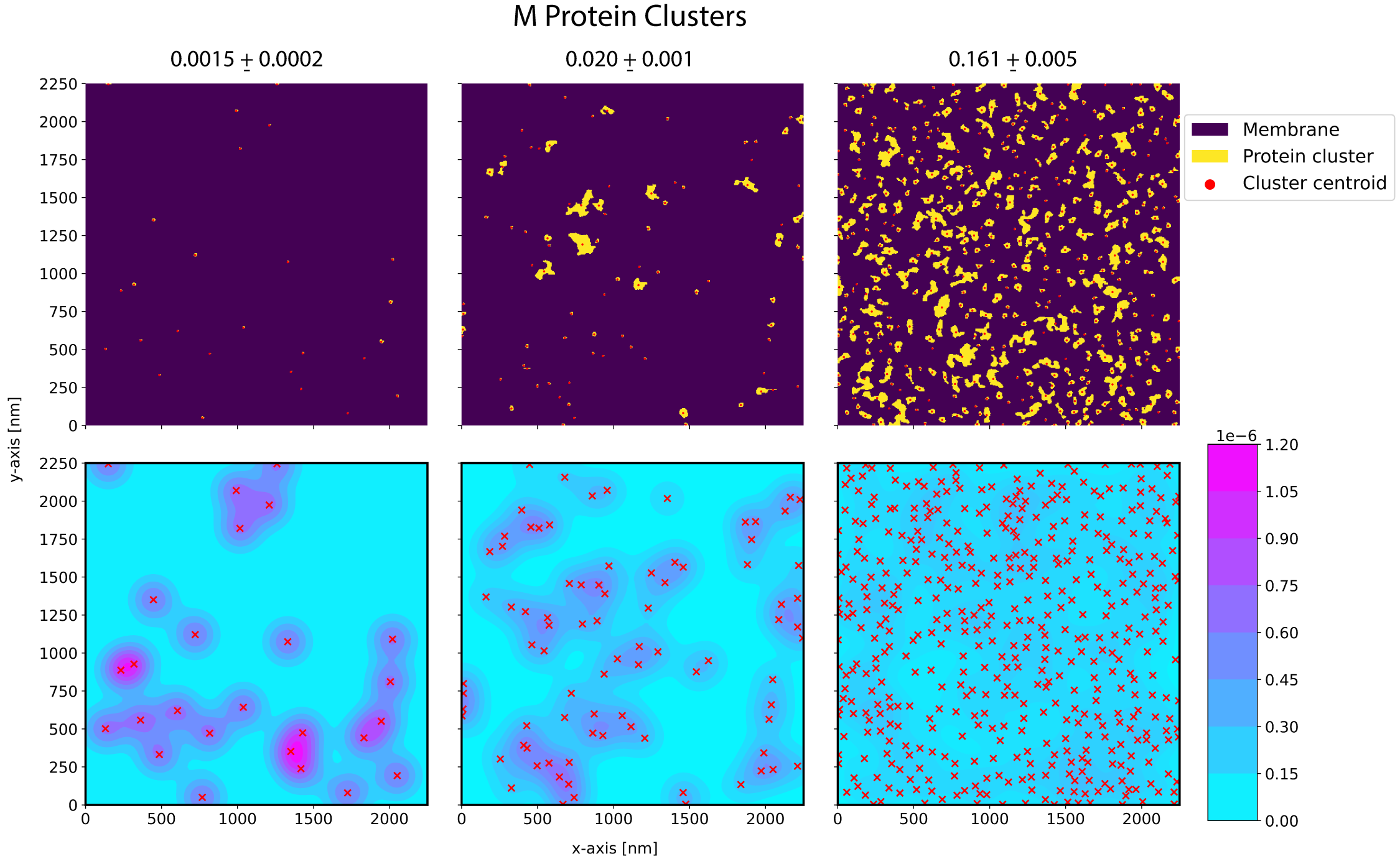

Supplement: S8 Fig — The top panel represents filtered and thresholded AFM data for each area coverage with cluster centroids shown as red dots. A cluster is defined as five or more adjacent pixels with height values greater than the threshold. Two-dimensional kernel density estimates of centroids are shown in the panel below, where higher density is shown in pink and lower is shown in blue. Of the three area coverages, only the highest has a close to constant density, showing isotropic cluster distribution. (TIF) [file pcbi.1014229.s010.tif]

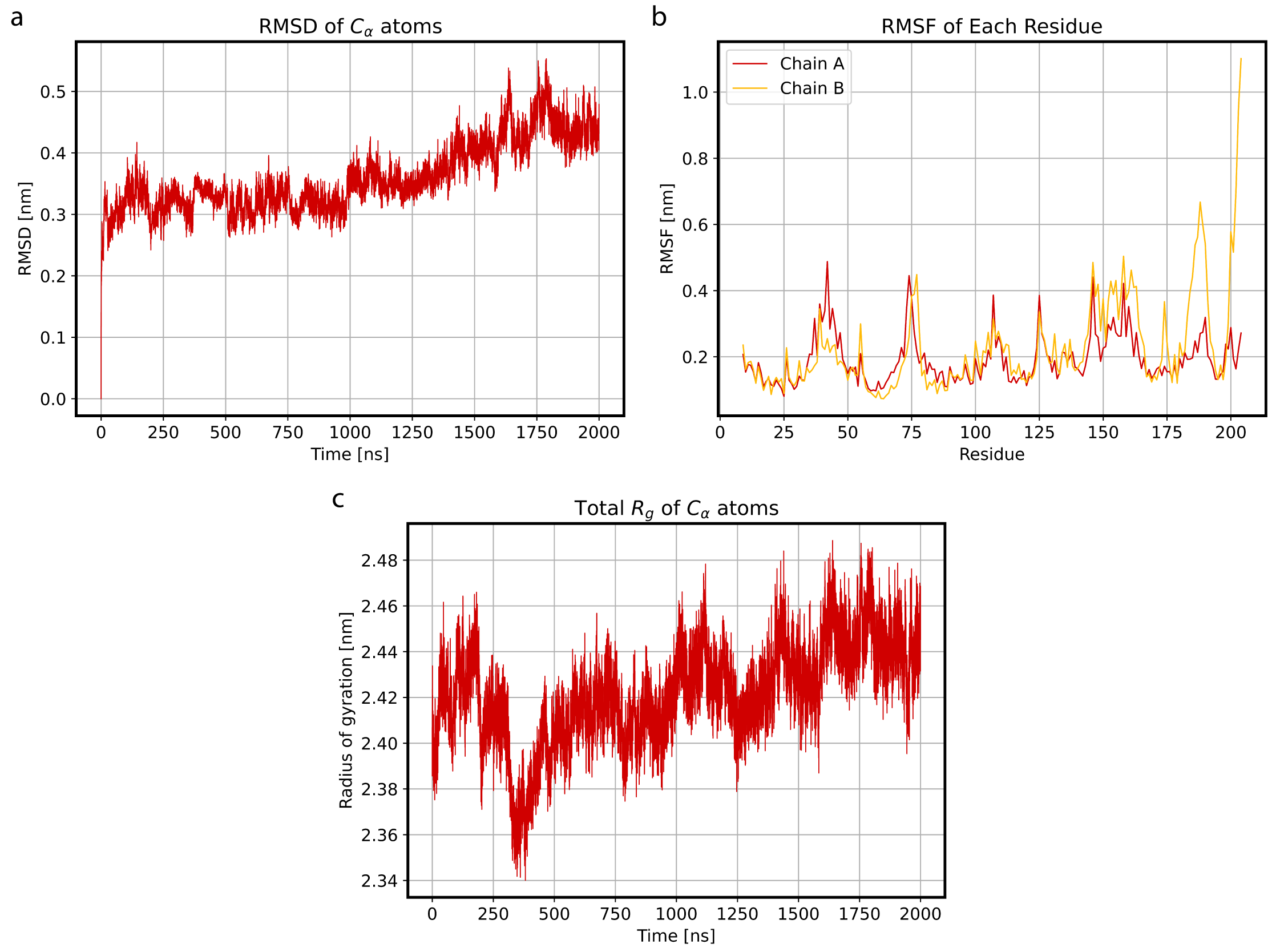

Supplement: S9 Fig — (a) Root mean square deviation (RMSD) defines how much a protein structure has changed relative to its initial position. It is defined as RMSD(t)=1N∑CαN[ri(t)−ri,0]2, where N is the total number of Cα atoms, ri(t) is the position for the i’th Cα atom at time t, and ri,0 is the initial position of the corresponding atom. (b) Root mean square fluctuation (RMSF) is shown for both short form chains, where the N-terminal starts at residue 9 and the C-terminal ends at residue 204. RMSF is the averaged RMSD over time for each residue. (c) Total radius of gyration (Rg) of short form Cα atoms is shown in red. With little change throughout the simulation in (a) and (c), and reasonable RMSF for each residue in (b), the protein remains stable throughout the simulation. All of these quantities were calculated using the appropriate GROMACS command. (TIF) [file pcbi.1014229.s011.tif]

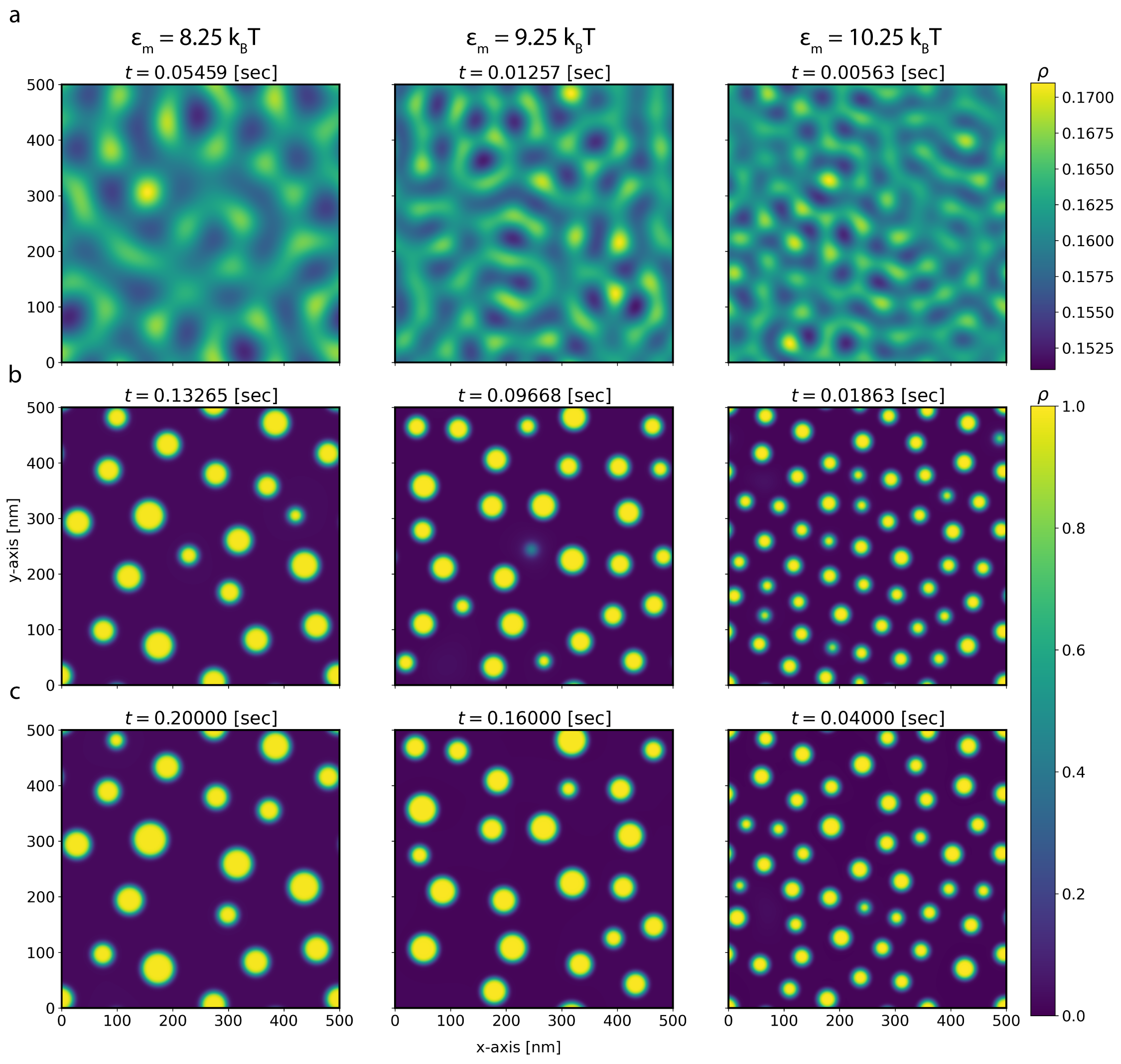

Supplement: S10 Fig — (a) Frames at the end of linearity from Fig 4a. (b) Frames before Ostwald ripening dominates from Fig 4b. (c) Final simulation frames for corresponding interaction energies. Slight dissipation of clusters can be seen in each final frame, where smallest clusters in (b) or weaker maxima in (a) dissipate. Times are shown at the top of each image. (TIF) [file pcbi.1014229.s012.tif]

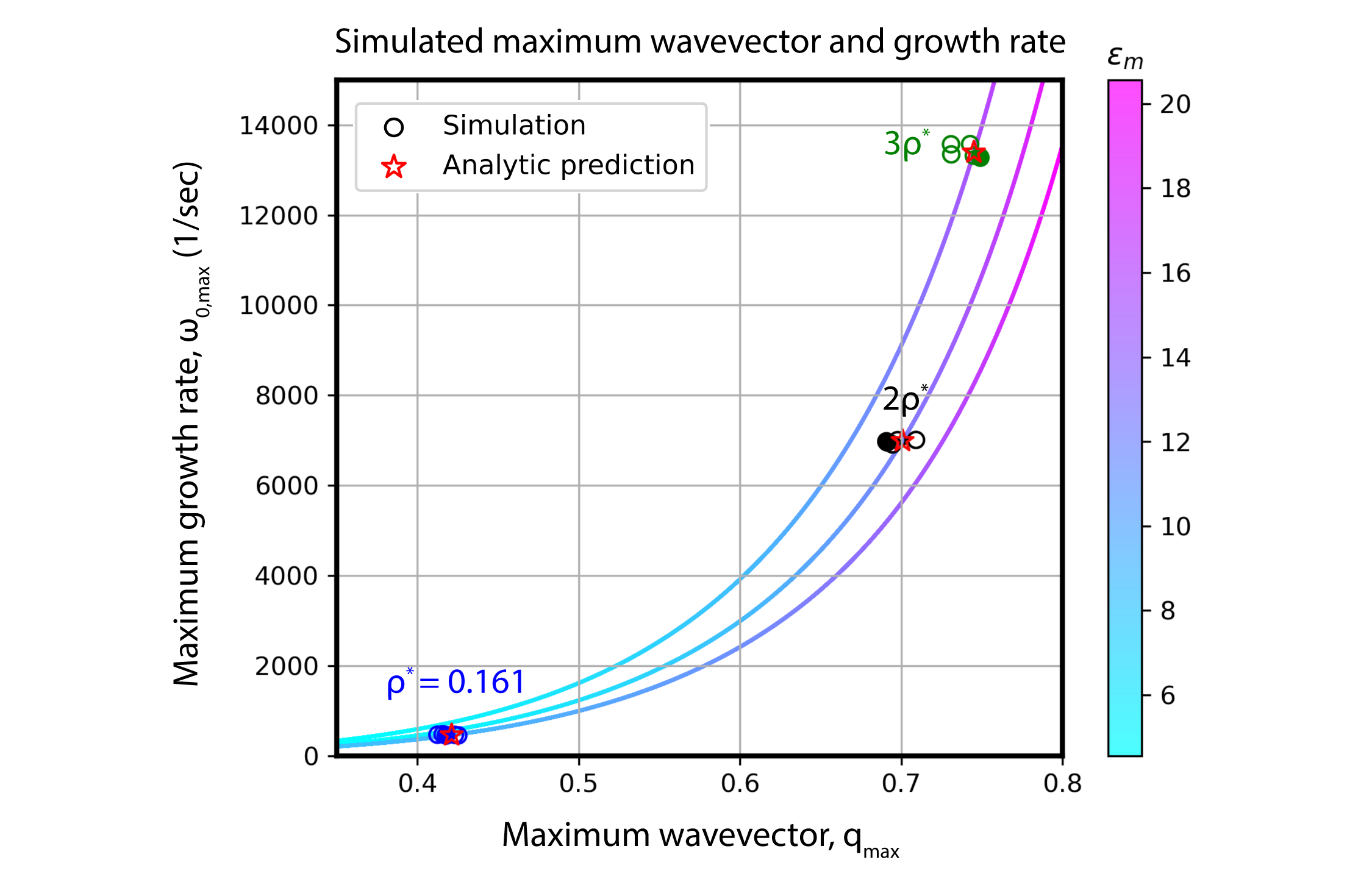

Supplement: S11 Fig — Each simulation was performed at ϵm=9 kBT, with five replicates for each of the three different initial protein densities: ρ*=0.161 (blue), 2ρ* (black), and 3ρ* (green). Filled in circles correspond to replicates shown in Fig 5. Analytical curves from Eq. 25 in S1 Appendix, where the color gradient represents different effective interaction energies, are shown for each initial density fraction. Red stars are the expected analytic value for the maximum wavevector and maximum growth rate dependent on ρ*. The lowest curve corresponds to ρ*=0.161 at a variety of effective interaction energies, while the middle applies to 2ρ*, and the upper 3ρ*. (TIF) [file pcbi.1014229.s013.tif]
