# Supplementary material for: Clustering of SARS-CoV-2 membrane proteins in lipid bilayer membranes
Source: PLoS Comput Biol. 2026 Apr 27;22(4):e1014229. doi: 10.1371/journal.pcbi.1014229 (PMC13148779; doi:10.1371/journal.pcbi.1014229)
Supplement: Attachment — Submitted filename: Revision_Letter_Final.pdf [file pcbi.1014229.s023.pdf]

We thank the reviewers for their careful reading of the manuscript and their extensive, insightful and constructive comments that have helped us greatly improve the quality of our manuscript. We have addressed each comment below and indicated (by line number of the marked-up manuscript) the corresponding changes in the main manuscript. The changes in the main manuscript are highlighted in color with red strikethrough for deletions and blue text for new additions. In addition to edits addressing reviewer comments, we also made minor grammatical changes throughout to improve flow and readability. Any citations included in this response follow the main text's numbering.

Reviewer #1:

This manuscript integrates long-timescale all-atom MD simulations, continuum modeling using a Cahn–Hilliard framework, and AFM imaging to quantify the interplay between direct M–M interactions and membrane-mediated forces in driving SARS-CoV-2 M protein clustering. The work aims to define the critical interaction energies and protein densities required for the onset of cluster formation, with implications for viral assembly mechanisms.

Overall, the study addresses a biologically important question and employs a rigorous multiscale approach. The manuscript is clearly written and technically sound, but certain aspects need clarifications, methodological justification, and better contextualization within coronavirus biology.

We thank the reviewer for these positive comments on our work.

Major:

1. While the physics-based treatment is elegant, the biological implications are underdeveloped. The conclusion that "direct M–M interactions dominate over membrane-mediated interactions" is quantitatively supported, but the structural basis of such interactions is not discussed. Structural models of M dimers (short vs long forms) and known oligomerization interfaces should be incorporated into the interpretation.

We are grateful for the reviewer's suggestion to improve the manuscript by incorporating discussion of structural models and oligomerization interfaces. Structural models of the M protein dimerization interface have been extensively classified, where both the long and short conformations act as homodimers with three domains – an extravirion N-terminal, an embedded transmembrane domain, and an intravirion C-terminal domain (lines 49-51) [28,35]. The dimerization interface involves contacts within the transmembrane and C-terminal domains – with the transmembrane domain potentially dominating [28,83,84]. While the higher-order oligomerization interface(s)

has not yet been identified, adjacent dimers tend to contact each other through the C-terminal domain [13,28], as needed for the observed tightly packed clusters. Additionally, higher-order oligomerization has been observed [13,28,36,41], providing support for our effective oligomerization energy (previously defined as the direct M-M interaction energy). Note that the lack of any well-defined dimer-dimer interface implies that our effective oligomerization energy quantifies a set of interfaces as opposed to a single one (lines 239-241). Although our effective oligomerization energy assumes dimer-dimer interactions, monomer-monomer contacts are also capable of playing a role. In addition to the new text in the lines quoted above, we also addressed the issue in detail in the discussion section through the addition of lines 605-623.

2. The manuscript should discuss how the estimated interaction energies (≈ 7.8–9.6 kBT) compare to known coronavirus M protein oligomerization data, and viral assembly thresholds in other enveloped viruses.

We thank the referee for this important suggestion. First, numerous experimental studies have confirmed the existence of higher-order oligomers [13,28,36,41]. All-atom molecular dynamics simulations of M protein structural predictions [83] reveal the monomer-monomer binding energy to be of similar scale to our estimated energies through potential of mean force calculations (lines 617-620). However, to our knowledge quantification of the oligomerization energy has not been performed. Additionally, we are unaware of any experimental studies quantitatively examining viral assembly thresholds in enveloped viruses – although we would greatly appreciate any suggestions. As a best alternative, we compared our estimated effective interaction energy – and line tension – to those measured for different transmembrane protein using high-speed AFM. We now include a more detailed reference to this comparison in the discussion (lines 601-604).

3. The discussion would benefit from a clearer explanation of whether the derived critical densities are physiologically plausible in ERGIC membranes.

This is indeed a very important point. We absolutely agree that comparing our predicted critical densities to physiologically plausible values, as highlighted by the reviewer, is desirable. Unfortunately, to our knowledge no experimental study has quantified M protein density – or that of other viral proteins – along the ERGIC during assembly. So, we decided to roughly estimate the density with a back of the envelope calculation using images of assembly and budding in compartments from infected cells.

Given that a single virion holds ~1000 M proteins [13], we assume that the number of virions forming in a compartment (n_v) defines the minimum M protein count currently on the surface. With each M protein having an approximate radius of $r_M = 2.5$ nm, the resulting area fraction for a closed spherical compartment with radius r_C is given by $250 n_v \left(\frac{r_M}{r_C}\right)^2$. Given the variance in compartment sizes and number of virions currently being produced [41,86-92], we estimate this area fraction for small [88] compartments (100 nm) holding a single virion and large [86] compartments (300 nm) holding 10 or more from differing studies. In both cases, we estimate an M protein area fraction lower bound of ~ 0.17. This lends plausibility to our predicted density range. We have added an explanation of this estimate in the discussion section (lines 628-638). Note that this serves as a lower bound because a compartment could have virions forming that we do not observe and not all M proteins are within forming virions. Additionally, we also simplified the complex shape of these compartments to a sphere.

Minor:

1. Recent cryo-EM studies on coronavirus M oligomeric states should be more thoroughly cited.

Thanks to the reviewer's suggestion, we have cited every recent cryo-EM study we are aware of that provides an M protein dimerization interface [28,35,**40,41,81,82**]. Bolded sources are newly added, where [40,41] gives M protein structures altered by small molecules, and [81, 82] reports structures for different coronaviruses. A discussion of these sources is added in lines 605-623.

Reviewer #2:

This manuscript presents an important multiscale investigation of SARS-CoV-2 M-protein clustering using all-atom MD, continuum Cahn–Hilliard modeling, and AFM imaging. The topic is timely, the methods are generally robust, and the results offer quantitative insight into M–M interactions during early viral assembly. The work has clear potential for publication.

We thank the reviewer for these positive comments on our work.

However, several issues require clarification before acceptance:

Continuum Model Assumptions:
The model neglects curvature–composition coupling despite the known curvature effects of M proteins (e.g., Introduction, lines 63–71). The authors should justify the flat-membrane approximation and discuss how it may shift critical interaction energies or densities.

We thank the reviewer for raising this important point. First, we emphasize that the motivation behind neglecting curvature induction in our model is the approximately flat nature of the supported lipid membrane in our AFM experiments and the absence of any observed curvature generation, thus enabling direct comparison between model and experiments. We have added text to emphasize this in several locations (in lines 82, 101-102, 178-180, 399, 557). We also added a sentence to explicitly clarify that curvature induction is not included in our estimated effective interaction energy (line 233-234). As such, we stress that this study is best related to early stages of assembly and budding (lines 82, 91, 534-536, 652).

Following the referee's point, in the discussion we now highlight how curvature can enhance cluster formation, increasing our effective interaction energy, and lowering the required thresholds for assembly (lines 642-647) [68,93,94]. Lastly, we note that whether M protein induces curvature responsible for virion formation remains to be seen – as any evidence of M protein curvature preference is indirect (lines 54-55). This is something we are actively working on.

Line Tension Estimation:
The thinning-induced line tension (~0.1 kBT/nm) relies on generic elasticity parameters rather than system-specific values. A sensitivity analysis or clearer limitations is needed.

This raises an important point – which we thank the referee for. To clarify, the original bending modulus was estimated from Young's modulus measurements of a similar M protein populated supported lipid bilayer system [36]. For simplicity we assume the tilt moduli to be of identical magnitude. However, accounting for standard deviation in these Young's modulus measurements, and conservatively estimating the resulting error, leads to $B_s \sim 3.0\ k_{\mathrm{B}}T \pm 1\ k_{\mathrm{B}}T$ and $K_s \sim 3.0\ k_{\mathrm{B}}T/\mathrm{nm}^2 \pm 1\ k_{\mathrm{B}}T/\mathrm{nm}^2$. This more robust quantification of our moduli is discussed in lines 160-164, and 366-367.

Additionally, this leads to a range for our line tension, $\gamma_m = 0.10\ k_{\mathrm{B}}T/\mathrm{nm} \pm 0.04\ k_{\mathrm{B}}T/\mathrm{nm}$ (lines 79, 368-369, 555, and 592-593). As a result, thinning's contribution to the effective interaction energy is now $[0.4\ k_{\mathrm{B}}T,\ 1.5\ k_{\mathrm{B}}T]$ – while also accounting for the protein's variable width (a). Since the contribution to ϵ_m is a range dependent on a,

the lower and upper bounds of ϵ_{olig} (previously $\epsilon_{\mathrm{direct}}$) have changed to $[6.9\ k_{\mathrm{B}}T, 8.9\ k_{\mathrm{B}}T]$. These changes can be seen in lines 102, 594, 601.

AFM Area Coverage Determination:
Protein coverage estimates depend heavily on threshold selection (S7 Fig). The lowest-density image uses a threshold borrowed from another dataset, which may bias results. A robustness check is recommended.

As pointed out by the referee, threshold selection is vital for determination of protein density. Thanks to this comment, we added insets to S7 Fig panel b to highlight how area coverage changes with slight threshold height alterations. The whole panel is included below, where the error in protein area fraction corresponds to how it changes when shifting threshold heights up and down by AFM vertical resolution. The caption for S7 Fig was altered accordingly (line 806-807), and we emphasize this alteration in the methods section (line 326-327). Note that we re-use the middle-density threshold on the lowest-density image because M protein density is most comparable between those two systems.

b

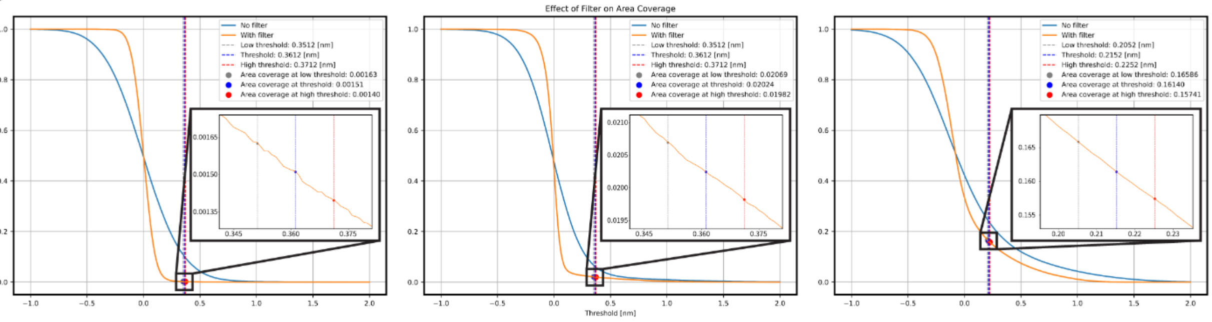

S7 Fig panel b: Protein area coverage is shown as a function of threshold height, where the used value is shown as a dotted vertical blue line. As the filter is applied, the curve gets closer to a step function, as expected for a transmembrane protein. The dotted grey line represents the higher bound on area coverage when considering AFM vertical resolution, while the dotted red line signifies the reverse, leading to the error bars in S7 Fig panel a. Insets highlighting the change in area coverage near the chosen threshold heights are displayed for each system.

Mismatch at Higher Densities:
Simulations at 2ρ* and 3ρ* show strong coarsening (Fig 5c–d), unlike AFM images. The authors should discuss whether biological membranes exhibit mechanisms that arrest phase separation, which are not included in the model.

We thank the referee for raising the issue of arrested phase separation. First, we clarify that the densities in simulations that display coalescence are significantly higher than our AFM experiments (lines 581-582). Additionally, as opposed to our simulations at late times, we do not see any evidence of Ostwald ripening in AFM images, indicating potentially kinetically arrested states. Thus, the referee is right that there could be factors that arrest phase separation our model is not accounting for.

A potential mechanism in our system is interactions between the M protein's N-terminal and the bilayer supporting mica surface, slowing down kinetics, as described in lines 564-566. Alternatively, inside the cell, local or even M protein spontaneous curvature can limit assembly sizes. We now refer to this aspect in lines 642-647.

Applicability of Linear Stability Analysis:
The interaction energy ϵm derived from AFM spacing uses a linear-regime expression (Eq. 15), while AFM patterns are fully nonlinear. The authors should justify this extrapolation or clarify its limitations.

This is an important aspect of our manuscript, and we are grateful to the reviewer for bringing this up. The simulations in Fig 4 confirm that this linear-regime ϵ_m is valid even beyond linearity in the diffusion limited growth regime – until Ostwald ripening starts taking over. As such, this relation can be applied to our AFM images if the density is low enough for coalescence to have little impact, and AFM patterns are kinetically arrested before Ostwald ripening begins to dominate. These limitations are now additionally emphasized in lines 508-509, and 587-589. We also address AFM patterns potentially being kinetically trapped in lines 564-566.

Despite these concerns, the manuscript is promising and would merit publication after major revision.

<u>Reviewer #3:</u>

General Assessment

The manuscript presents a well-designed multiscale study combining molecular dynamics simulations, continuum modeling, and AFM experiments to examine clustering of the SARS-CoV-2 membrane (M) protein. The approach is technically

sound, and the quantitative estimates of effective M–M interaction energies are a clear strength. The study addresses an important question in viral assembly and fits well within the scope of PLOS Computational journals. The main conclusions are supported by the data, but clearer framing of the biological scope is needed to avoid overgeneralization.

We thank the reviewer for these positive comments on our work.

Major Comments

1. Biological Context and Scope

The study focuses exclusively on the short-form M protein dimer, while structural studies indicate that a long-form conformation is associated with membrane curvature and later stages of budding. The authors should clearly state that their conclusions are most relevant to early-stage assembly on relatively flat membranes. A short discussion of whether the estimated interaction energies could stabilize the long form, and how E or N proteins might influence clustering, would improve biological clarity.

As helpfully pointed out by the reviewer, these results are most relevant to the onset of assembly and budding – as the membrane is flat in both AFM data and our model. To address this, we alter lines 82, 91, 399, 534-535, 557, and 651-655. Additionally, interactions between M/E proteins [15,31,95] and M/N proteins with RNA [13,28,30,82] are potentially capable of enhancing clustering by lowering critical thresholds. Lines 647-651 are added to describe this point. Lastly, thanks to the reviewer's comment, we highlight the potential for this interaction energy's ability to stabilize the long form, while mentioning other possible causes for the long/short transition (line 660-662).

2. Interpretation of Direct Interaction Energy

The direct interaction energy (ϵ_direct ≈ 6–8 kBT) should be described as an effective oligomerization energy, rather than as a single structural interface. This clarification will help readers interpret the parameter appropriately.

We appreciate the referee's important point – and clarify that the previously labeled 'direct interaction energy' serves as the energy cost in drawing a dimer to another dimer. Furthermore, the exact interface two M protein dimers make is unknown, and thus our parameter describes a set of different interfaces. All of this means that 'direct interaction energy' is better described as an 'effective oligomerization energy' (ϵ_{olig}) as rightly pointed out by the referee. This modification is made when appropriate

throughout the manuscript, starting with the brief addition of “through an effective oligomerization energy” in the abstract, and continuing with lines 101-102, 239-241, 243, 597-601, 606, 615-617, 738, and Eq. 13, 30, and 31.

3. Simplified Protein System

The simulations exclude the C-terminal cytosolic tail of M and other viral components (N, E, RNA). The authors should note that the reported interaction energies likely represent a lower bound for in vivo conditions and that additional components are expected to influence cluster stability and later assembly steps.

The referee is absolutely right. The inclusion of other viral components will lower the critical effective interaction energy and protein density due to attractive interactions between M/E proteins [15,31,95] and M/N proteins with RNA [13,28,30,82]. Note that rather than altering our effective interaction energy, adding components along the surface will lead to additional interaction energy parameters [42,43]. As such, we address the critical quantity shift in lines 647-651, and the influence these additional behaviors have on later budding stages in lines 651-655.

Lastly, as recognized by the referee, the M protein short form structure utilized in our simulations excludes residues 1-8 and 205-222 [28]. This exclusion could bias the thinning behavior identified in our simulation, altering the resulting ϵ_{olig}. However, our simulated thinning profile remains in agreement with AFM images of individual, full-length M proteins [36], implying these residues do not substantially alter the resulting line tension. Since the full M protein is used in gathering our AFM data, this point does not alter ϵ_m. Lines 550-552 were altered to address the similarity between AFM profiles of complete M proteins, and our simulations with excluded residues.

Minor Comments

1. Clarify the physical meaning of the length scale a.

Beyond serving as a length scale, a is the nearest neighbor distance in a protein lattice – describing how tightly M proteins are packed. Since we observe tight packing in our AFM images, this distance is taken to be the approximate width of an M protein dimer (S2 Fig). It can also define the M protein saturation density $\rho_s = \frac{1}{a^2}$. This comment was addressed in lines 187-189, 258, 507, 594, 719, and 747.

2. Briefly contextualize the interaction energy scale using known membrane protein oligomerization energies.

Comparison of our interaction energies with other transmembrane proteins serves as a great way to enhance the paper. In the original manuscript we briefly mentioned the energy scale of a different transmembrane protein without sufficient detail. Now, we highlight this study, which used high-speed AFM imaging to measure the interaction energy of a model transmembrane protein. They found membrane thinning introduced a $1 - 2\ k_{\mathrm{B}}T$ attraction out of a total interaction of $\sim 6\ k_{\mathrm{B}}T$, similar in scale to both our energies. This point is made in lines 601-604.

3. Improve figure readability where possible.

To accommodate the referee's comment, we improved the readability of S7 Fig, including insets to highlight the slight change in area coverage given local alterations in threshold. Furthermore, we changed the color of the $\epsilon_m = 9\ k_B T$ data points in Fig. 4c and S11 Fig to blue for visibility. Note that we also updated the equation shown in Fig 4d, as the equation in the original version was missing a factor of $k_B T$ (Eq. 16). Below, we compare altered figures to their original versions.

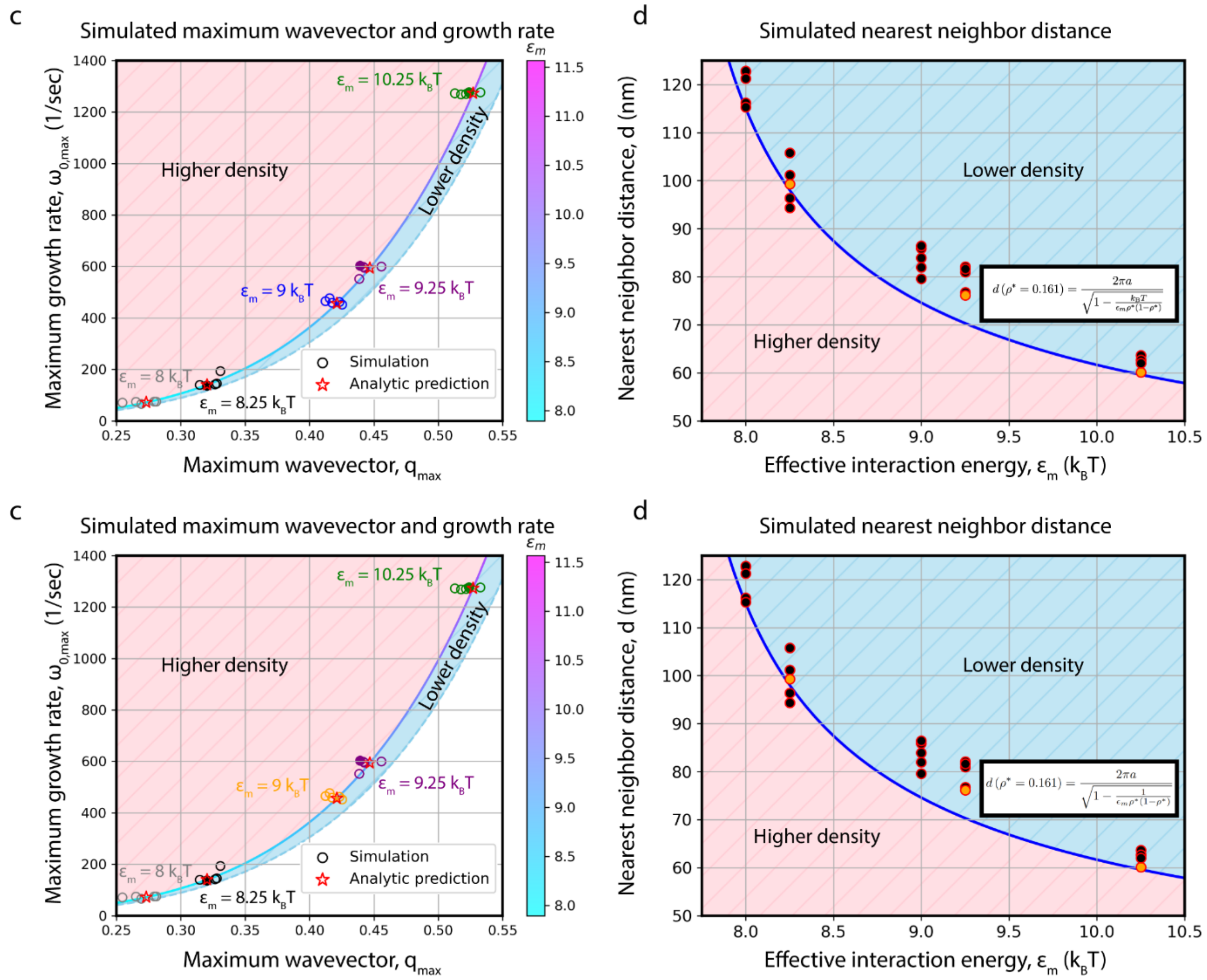

Fig 4c and d: Changes made for Fig 4c and d, where the top is new and the bottom is old.

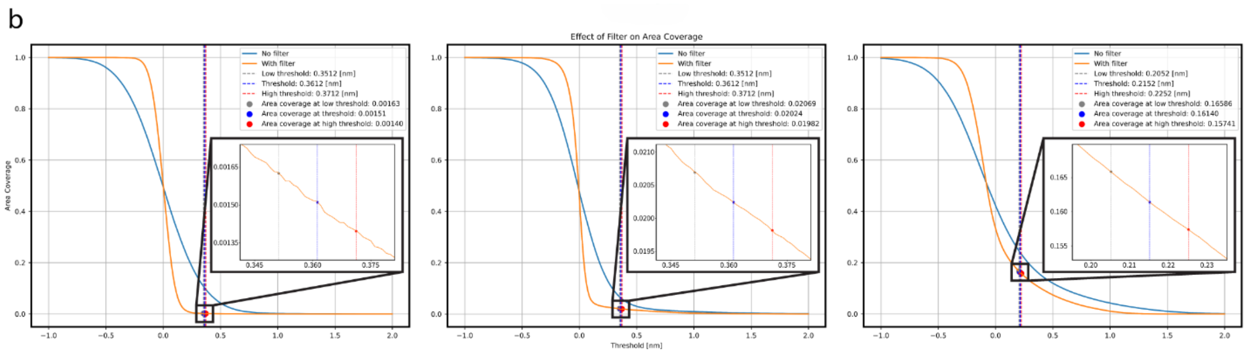

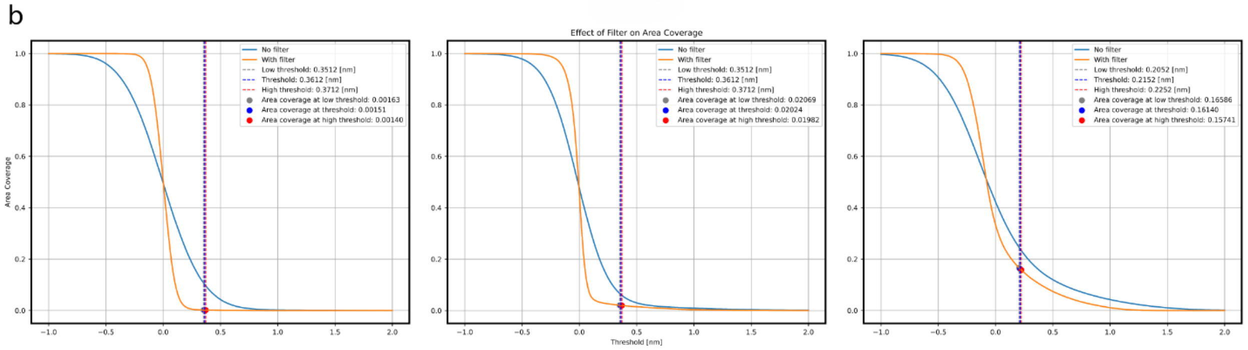

S7 Fig panel b: Changes made for S7 Fig panel b, where the top is new and the bottom is old.

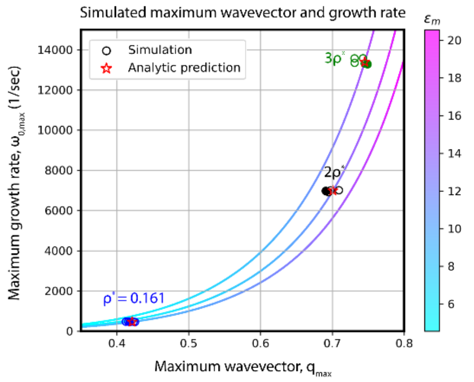

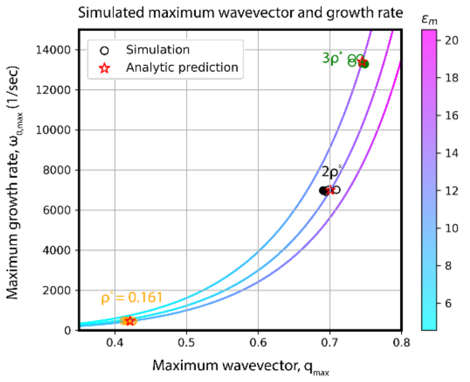

S11 Fig: Changes made for S11 Fig, where the left is new and the right is old.

Recommendation

Major Revision
