# Supplementary material for: Clustering of SARS-CoV-2 membrane proteins in lipid bilayer membranes
Source: PLoS Comput Biol. 2026 Apr 27;22(4):e1014229. doi: 10.1371/journal.pcbi.1014229 (PMC13148779; doi:10.1371/journal.pcbi.1014229)
Supplement: Attachment — Submitted filename: Reviewer_Report_PLOSONE Comp.docx [file pcbi.1014229.s021.docx]

**Reviewer Report**

**General Assessment**

The manuscript presents a well-designed multiscale study combining molecular dynamics simulations, continuum modeling, and AFM experiments to examine clustering of the SARS-CoV-2 membrane (M) protein. The approach is technically sound, and the quantitative estimates of effective M–M interaction energies are a clear strength. The study addresses an important question in viral assembly and fits well within the scope of PLOS Computational journals. The main conclusions are supported by the data, but clearer framing of the biological scope is needed to avoid overgeneralization.

**Major Comments**

**1. Biological Context and Scope**

The study focuses exclusively on the short-form M protein dimer, while structural studies indicate that a long-form conformation is associated with membrane curvature and later stages of budding. The authors should clearly state that their conclusions are most relevant to early-stage assembly on relatively flat membranes. A short discussion of whether the estimated interaction energies could stabilize the long form, and how E or N proteins might influence clustering, would improve biological clarity.

**2. Interpretation of Direct Interaction Energy**

The direct interaction energy (ϵ_direct ≈ 6–8 kBT) should be described as an effective oligomerization energy, rather than as a single structural interface. This clarification will help readers interpret the parameter appropriately.

**3. Simplified Protein System**

The simulations exclude the C-terminal cytosolic tail of M and other viral components (N, E, RNA). The authors should note that the reported interaction energies likely represent a lower bound for in vivo conditions and that additional components are expected to influence cluster stability and later assembly steps.

**Minor Comments**

1. Clarify the physical meaning of the length scale a.

2. Briefly contextualize the interaction energy scale using known membrane protein oligomerization energies.

3. Improve figure readability where possible.

Recommendation

Major Revision
